# The variation of acute treatment costs of trauma in high-income countries

**DOI:** 10.1186/1757-7241-21-S1-S6

**Published:** 2013-05-28

**Authors:** L Willenberg, K Curtis, C Taylor, S Jan, P Glass, J Myburgh

**Affiliations:** 1 The George Institute, Sydney, Australia

## Background

In order to assist health service planning, understanding factors that influence higher trauma treatment costs is essential. [1] The majority of trauma costing research reports the cost of trauma from the perspective of the receiving hospital. There has been no comprehensive synthesis and little assessment of the drivers of cost variation, such as country, trauma, subgroups and methods. The aim of this review is to provide a synthesis of research reporting the trauma treatment costs and factors associated with higher treatment costs in high income countries.

## Methods

A systematic search for articles relating to the cost of acute trauma care was performed and included studies reporting injury severity scores (ISS), per patient cost/charge estimates; and costing methods. Cost and charge values were indexed to 2011 cost equivalents and converted to US dollars using purchasing power parities.

## Results

A total of twenty-seven studies were reviewed. Eighty-one percent of these studies were conducted in high income countries including USA, Australia, Europe and UK. Studies either reported a cost (74.1%) or charge estimate (25.9%) for the acute treatment of trauma. Across studies, the median per patient cost of acute trauma treatment was $22,448 (IQR: $11,819-$33,701). However, there was variability in costing methods used with 18% of studies providing comprehensive cost methods. Sixty-three percent of studies reported cost or charge items incorporated in their cost analysis and 52% reported items excluded in their analysis. In all publications reviewed, predictors of cost included Injury Severity Score (ISS), surgical intervention, hospital and intensive care, length of stay, polytrauma and age.

## Conclusion

The acute treatment cost of trauma is higher than other disease groups. Research has been largely conducted in high income countries and variability exists in reporting costing methods as well as the actual costs. Patient populations studied and the cost methods employed are the primary drivers for the treatment costs. Targeted research into the costs of trauma care is required to facilitate informed health service planning.

## References

[B1] Thomson S Addressing financial sustainability in health systems 2009 Denmark: European Observatory on Health Systems and Policies

